# Cerebral blood flow and cognitive functioning in patients undergoing transcatheter aortic valve implantation

**DOI:** 10.1016/j.eclinm.2025.103092

**Published:** 2025-02-14

**Authors:** Astrid C. van Nieuwkerk, Kimberley I. Hemelrijk, Hugo M. Aarts, Anna E. Leeuwis, Charles B.L.M. Majoie, Mat J.A.P. Daemen, Esther E. Bron, Justine E.F. Moonen, Alexandra de Sitter, Berto J. Bouma, Alexander Harms, Wiesje M. van der Flier, Jan Baan, Jan J. Piek, Geert Jan Biessels, Ronak Delewi, Heart-Brain Connection Consortium

**Affiliations:** a Department of Cardiology, Amsterdam Cardiovascular Sciences, Amsterdam UMC, University of Amsterdam, Amsterdam, the Netherlands; b Alzheimer Center Amsterdam, Neurology, Vrije Universiteit Amsterdam, Amsterdam UMC location VUmc, Amsterdam, the Netherlands; c Amsterdam Neuroscience, Neurodegeneration, Amsterdam, the Netherlands; d Old Age Psychiatry, GGZ inGeest, Amsterdam, the Netherlands; e Department of Radiology and Nuclear Medicine, Amsterdam Neurosciences, Amsterdam UMC, University of Amsterdam, Amsterdam, the Netherlands; f Department of Pathology, Amsterdam University Medical Center, Locations AMC and VUmc, University of Amsterdam, Amsterdam, the Netherlands; g Biomedical Imaging Group Rotterdam, Department of Radiology & Nuclear Medicine, Erasmus MC - University Medical Center Rotterdam, Rotterdam, the Netherlands; h Epidemiology and Data Science, Vrije Universiteit Amsterdam, Amsterdam UMC location VUmc, Amsterdam, the Netherlands; i Department of Neurology, UMC Utrecht Brain Center, University Medical Center, Utrecht, the Netherlands

**Keywords:** Aortic valve stenosis, Transcatheter aortic valve replacement, Cerebrovascular circulation, Cognition, Hemodynamics, Neuropsychological tests, Magnetic resonance imaging

## Abstract

**Background:**

Approximately one-third of patients with symptomatic severe aortic valve stenosis scheduled for transcatheter aortic valve implantation (TAVI) have some degree of cognitive impairment. The effect of TAVI on cardiac output, cerebral blood flow (CBF), and cognitive functioning has not been systematically studied.

**Methods:**

CAPITA (NCT05481008) is a prospective longitudinal study assessing cerebral and cognitive outcomes in patients that underwent TAVI between August 2020 and October 2022. At baseline (<24 h before TAVI) and three-month follow-up, patients underwent echocardiography, brain magnetic resonance imaging (MRI), and multidomain neuropsychological assessment. Primary outcome measures were change in CBF (Δml/100 g/min on arterial spin labelling MRI) and change in global cognitive functioning (Δz-scores). Secondary outcomes included cardiac output (L/min), and white matter hyperintensities (mL, number). Differences were tested with paired t-test and associations were tested with linear mixed models.

**Findings:**

A total of 148 patients (80.5 ± 5.7 years, 43% female) underwent TAVI. Three months after TAVI, cardiac output increased from 5.9 ± 1.4 L/min to 6.3 ± 1.4 L/min (mean difference 0.37, 95% CI 0.12–0.62, p = 0.004). CBF increased from 52.2 ± 14.5 mL/100 g/min to 55.9 ± 17.7 mL/100 g/min (mean difference 3.8, 95% CI 1.15–6.36, p = 0.005). Global cognitive functioning also increased from 0.02 ± 0.52 to 0.15 ± 0.49 (mean difference 0.13, 95% CI 0.06–0.20, p < 0.001) with most prominent increase in patients with worst baseline cognitive functioning. Patients with cognitive decline (22%), had a higher volume of new in white matter hyperintensities than patients with stable or improved cognition (78%): 1.26 ± 2.96, vs 0.29 ± 0.45, vs 0.31 ± 0.91 mL (p = 0.06).

**Interpretation:**

In patients with severe symptomatic aortic valve stenosis undergoing TAVI, cardiac output, CBF, and cognitive functioning improved after three months.

**Funding:**

The Heart-Brain Connection crossroad consortium of the 10.13039/100018890Dutch Cardiovascular Alliance. The Netherlands CardioVascular Research Initiative: 10.13039/100002129Dutch Heart Foundation (CVON 2018-28 & 2012-06 Heart Brain Connection).

**Research in context**

**Evidence before this study**

Previous studies in patients with severe aortic valve stenosis undergoing transcatheter aortic valve implantation (TAVI) have shown that TAVI induces cerebral emboli on magnetic resonance imaging (MRI) in three quarters of patients. The high rates of cerebral infarcts induced concerns about cognitive harm of TAVI. However, most patients do not experience symptoms of these cerebral lesions. Preliminary studies are contradictory whether TAVI, possibly through these cerebral lesions, may have negative effects on cognitive functioning. However, some studies have shown improved cognitive functioning in patients undergoing TAVI. We performed a systematic search on September 4, 2023, of PubMed, MEDLINE, and Embase using the terms “cerebral blood flow”, “brain perfusion”, “cerebral perfusion”, and “aortic valve stenosis”, “transcatheter aortic valve implantation”, “transcatheter aortic valve replacement”. Two pilot studies (n = 31, and n = 15) suggest potential improvement in cerebral blood flow following TAVI and one study (n = 27) after cardiac surgery. However, a recent TAVI study (n = 42) did not find improved CBF after the procedure. Nevertheless, all four studies were underpowered and no study systematically assessed cerebral blood flow and cognitive functioning in patients undergoing TAVI.

**Added value of this study**

For the first time, we show that cerebral blood flow, as measured on arterial spin labelling MRI, increases three months after TAVI. Importantly, cognitive functioning, as assessed with a comprehensive multi domain neuropsychological assessment improves in one third of these patients and cognitive functioning was stable in half of patients. In general, CAPITA reassures that TAVI is not harmful for cognitive functioning. Moreover, patients with low baseline cognitive functioning tended to have the largest post procedural increase in cognitive functioning. However, one in five patients had cognitive decline. Higher volumes of new white matter hyper intensities on follow-up MRI correlated with cognitive decline.

**Implications of all the available evidence**

This is the first large scale study showing that cognitive functioning is not only preserved, but may actually improve after TAVI in a subset op patients. Despite known evidence for procedural cerebral embolization, this unique study shows that TAVI has the potential to improve cognitive functioning and is not harmful for cognition in most patients. In daily clinical practice, CAPITA may reassure that patients with baseline cognitive impairment can be safely treated with TAVI with potential for cognitive improvement. These novel results can impact society as there is now evidence that cardiac interventions may benefit the brain. White matter hyperintensities may cause cognitive deterioration but this should be further assessed in future trials.

## Introduction

Severe aortic valve stenosis has a poor prognosis and will ultimately lead to functional deterioration and death if left untreated.^1^^,^^2^ In addition to physical symptoms, one third of patients with symptomatic severe aortic valve stenosis have some degree of cognitive impairment.^3^, ^4^, ^5^ Transcatheter aortic valve implantation (TAVI) is a life-saving treatment for patients with severe aortic valve stenosis, with a continuously expanding indication towards lower risk patients.^6^ However, stroke is a feared complication of TAVI.^7^ Up to 76% of patients have some extent of cerebral infarcts on postprocedural magnetic resonance imaging (MRI).^8^^,^^9^ High rates of cerebral infarcts induced concerns about cognitive harm of TAVI, but previous studies failed to show a direct relation between acute silent cerebral infarcts and cognitive decline.^8^^,^^9^ Most acute infarcts spontaneously resolve over time, and only a quarter remain visible as white matter hyperintensities (WMH) on longer term MRI and may negatively affect cognition.^9^^,^^10^ Cognitive preservation is especially important in light of expanding TAVI treatment indication.

Remarkably, a subset of patients undergoing TAVI have improved cognitive functioning after the procedure.^3^^,^^5^^,^^11^ Severe aortic valve stenosis substantially limits cardiac output. Potentially, to such a high degree that cardiac output provides insufficient cerebral perfusion pressure resulting in impaired cerebral blood flow (CBF).^12^^,^^13^ Cognitive improvement after TAVI may be due to improved cardiac and cerebral haemodynamics, however this hypothesis has not been systematically evaluated. We therefore hypothesize that TAVI may improve cardiac output, CBF, and cognitive functioning in a subset of patients. Simultaneously, we hypothesize that new WMH have adverse effects on cognitive functioning. Accordingly, the aim of this study was to systematically evaluate change in cardiac output, CBF, WMH, and cognitive functioning in patients undergoing TAVI.

## Methods

### Study design

The prospective longitudinal CAPITA (CArdiac outPut, cerebral blood flow and cognition In patients with severe aortic valve stenosis undergoing Transcatheter Aortic valve implantation) study assessed the effects of TAVI on cardiac output, CBF, and cognitive functioning. The study design with pre-specified endpoints has been described previously.^14^ At baseline and at three-month follow-up, patients underwent echocardiography (to determine cardiac output and valve functioning), 3 T MRI (arterial spin labelling [ASL] to assess CBF, T2 Fluid Attenuated Inversion Recovery [FLAIR] to assesses WMH and other structural lesions), and extensive neuropsychological testing. Patients served as their own controls: baseline measurements in presence of severe aortic valve stenosis were compared with post-TAVI measurements at follow-up. The study was conducted in accordance with the Declaration of Helsinki and followed the Strengthening the Reporting of Observational Studies in Epidemiology (STROBE) guidelines. The Institutional Review Board of the Amsterdam UMC approved the study (2019_802). All patients provided written informed consent. The study is registered at ClincalTrials.gov (NCT05481008).

### Objectives

The primary outcome measures of this study were change in global CBF (Δ mL/100 g/min) on ASL-MRI and change in global cognitive functioning (Δ z-score based on baseline measurements) between baseline and three-month follow-up.

Secondary outcome measures included change in cardiac output (L/min), change in WMH (mL, number), and associations between outcome measures. Differences in cardiac output were measured in ΔL/min on echocardiography. We assessed the incidence of new WMH in volume (Δ mL) and number on FLAIR MRI. Eventually, associations between cardiac output, CBF, and cognitive functioning were evaluated, as well as associations between new WMH and cognitive functioning. Clinical outcomes were defined according to the third Valve Academic Research Consortium,^15^ and neurological outcomes according to NeuroARC.^16^

### Patient selection

The prospective CAPITA study was performed in the Amsterdam UMC, a tertiary high-volume TAVI center. All patients with severe aortic valve stenosis planned to undergo TAVI between August 2020 and October 2022 were screened for study inclusion. Patients were approached for inclusion at the outpatient clinic of the Amsterdam UMC. Most important exclusion criteria were contra-indications for MRI and insufficient mastery of the Dutch language. Supplementary Table S1 presents all inclusion and exclusion criteria.

Indication for TAVI and selection of valve type, valve size, and access were made by a multidisciplinary heart team.^6^ Transfemoral access under local anaesthesia was the default approach, however if contra-indicated transaortic access was performed. Patients were treated with balloon-expandable Sapien 3 or Sapien 3 Ultra devices (Edwards Lifesciences, Irvine, USA); self-expandable Navitor (Abbott, Abbott Park, Illinois, USA) or Evolut R (Medtronic, Minneapolis, USA) devices. Predilatation and postdilatation were performed at operators’ discretion. Cerebral protection devices were not used.

### Measurement of outcomes

#### Echocardiography

Cardiac output was assessed by Doppler measurements of the left ventricular outflow tract velocity time integral on transthoracic echocardiography. This method has been validated against thermodilution, pulmonary artery catheter measurements and cardiac MRI.^17^, ^18^, ^19^ Transthoracic echocardiography (GE medical systems, Horten, Norway) was performed by trained echocardiographists before TAVI, immediately after TAVI, and at three-month follow-up, according to the European Association for Cardiovascular Imaging guidelines.^20^ Accordingly, aortic valve gradient, aortic valve area, ventricular function, and other valve pathologies were assessed. Echocardiographs were evaluated in a core laboratory using an automatic segmentation method and were visually checked by two independent reviewers who were blinded to clinical data and outcomes.

#### Brain MRI

All brain MRIs were performed on the same 3 T MRI scanner (Ingenia, Philips, Best, the Netherlands) using a standardized MRI protocol.^14^^,^^21^ CBF was defined as the volume of blood that flows through 100 g grey matter per minute. ASL-MRI measures global and regional CBF by use of an endogenous tracer.^22^ Methodological developments have increased its reproducibility and outcomes resemble those of positron emission tomography.^22^^,^^23^ It is the least invasive assessment method for CBF as it does not require intravenous contrast nor radiation.^23^ The scanner was equipped with a 16-channel DStream Head-Spine Coil and foam padding to restrict head motion. The pseudo-continuous ASL sequence was performed with a gradient-echo single-shot echo-planar imaging readout (matrix size = 80 × 80 voxels, voxel-size = 3.0 × 3.0 mm, 19 axial slices with 7.0 mm thickness without a slice-gap, echo time/repetition time = 17/4445 ms, SENSE = 2.5, initial post-label delay = 1800 ms; slice readout time = 35 ms; resulting post-label delay range for 19 slices = 1800–2465 ms, labelling duration = 1800 ms). Forty control–label pairs were acquired for each scan with a total scan duration of 4:44 min. The labelling plane was positioned 90 mm inferior parallel to the anterior-commissure–posterior-commissure line. ASL mapping was based on T1-weighted scans. ASL data was quantified into CBF based on a single-compartment model after the subtraction of labelled from control images. A subsequently acquired proton density-weighted image (M0) was used to scale signal intensities of subtracted ASL measurements to absolute CBF units, i.e., the volume of blood that flows through 100 g grey matter per minute (mL/100 g/min). ASL-MRI analyses were performed in an independent core laboratory using pipelines, which was blinded to clinical data. Raw ASL analyses were corrected for partial volume effects, motion correction, and brain volume.^24^^,^^25^ To minimize the effect of physiological confounders, time of day during scanning, room temperature and visuo-auditory stimuli were similar during baseline and follow-up. Patients had to abstain from caffeine for 3 h and from smoking and alcohol for at least 12 h.^23^ Haematocrit did not change between baseline and follow-up in our pilot study,^12^^,^^14^ therefore CBF was not corrected for haematocrit. Change in grey matter volume between visits may influence CBF and was therefore measured. WMH were defined as signal abnormalities of presumed vascular origin in the white matter on T2-weighted imaging.^21^ These are manifestations of cerebral small vessel disease or chronic infarction and have been associated with cognitive impairment.^26^ WMH were quantified using Lesion segmentation toolbox, an automated segmentation method with use of 3D T2 FLAIR sequences.^27^ Using these segmentations, we computed WMH number and volumes (mL). Cerebral injury was defined according to the NeuroARC composite endpoints of central nervous system infarction and haemorrhage: any infarction or haemorrhage on the basis of imaging, pathology, or clinical symptoms.^16^

#### Neuropsychological assessment

We assessed cognitive functioning with an extensive, standardized hour-long neuropsychological assessment.^28^ Neuropsychological testing was performed and scored by the same trained clinical neuropsychologist blinded to previous cognitive scores and imaging findings. Educational status was assessed according to the Verhage classification.^29^ For cognitive screening, we performed the Montreal cognitive assessment (MoCA) and mini-mental state examination (MMSE).^30^^,^^31^ The neuropsychological assessment covers global cognitive functioning based on four major cognitive domains: attention/psychomotor speed, memory, language, and executive functioning, each of which are presented separately.^14^^,^^28^ It also includes questionnaires for quality of life, depression, and apathy.^14^^,^^32^, ^33^, ^34^ The neuropsychological assessment included four cognitive domains. The cognitive tests included: trail making test part A and B,^35^ Stroop colour word test,^36^^,^^37^ letter digit substitution test,^38^ digit span forward and backward,^39^ 15-word auditory verbal learning test,^36^^,^^40^ and visual association test.^41^ Supplementary Table S2 lists all cognitive tests. Familiarisation trials were not used. To minimize learning effects and test-retest bias, we used a parallel version of the verbal learning test at follow-up measurements. Cognitive test scores were standardized into z-scores: (test score–mean baseline score)/standard deviation. Baseline and follow-up z-scores were based on baseline cognitive test scores for each patient. Higher z-scores represent better cognitive functioning. Cognitive domain scores were constructed as mean z-scores from all tests for the corresponding domain. Global cognitive function was calculated as the mean from the four domain scores. Cognitive improvement or decline was defined as >0.5 standard deviation (SD) change according to NeuroARC.^16^ Patients who died or were lost to follow-up due to reasons potentially associated with cognitive decline were added to the group of cognitive decline.

#### Statistical analysis

We tested baseline patient characteristics, cardiac output, CBF, and cognitive z-scores for normality with Q–Q plot and Shapiro–Wilk test. Accordingly, median with interquartile range (IQR) or mean ± SD were reported. Categorical variables were reported as frequencies and percentages. Global CBF as well as z-scores of global and domain-specific cognitive scores at baseline and three-month follow-up were compared using a paired t-test and reported as mean ± SD. Differences in CBF and z-scores were reported as mean difference (lower—upper 95% confidence interval [CI]). These analyses were not adjusted for age, sex, or education because subjects served as their own controls. Subjects were divided into equal tertiles of baseline cognitive functioning, and change in cognitive functioning was tested with one-way analysis of variance. Longitudinal differences for not normally distributed variables, such as WMH volume and number, were tested with Wilcoxon signed rank test and reported as median with IQR. The association between change in cardiac output (Δ L/min), CBF (Δ mL/100 g/min), and global cognitive functioning (Δ z-score) were assessed with linear mixed models. The model included age, sex, and education to correct for potential confounding. Cross-sectional associations at baseline or follow-up were assessed with linear regression. Results of regression models were reported as correlation coefficient (B) with 95% CI. The supplementary methods present the sample size calculation. Besides a new MRI contra-indication, there were no contra-indications for follow-up. All statistical tests were two-tailed and p < 0.05 was considered statistically significant. Analyses were calculated using SPSS (version 28.0 for Windows, SPSS, Inc., Chicago, USA). AN, KH, and RD had full access to the data.

### Role of funding

The funders of the study had no role in the study design, data collection, analysis, data interpretation, writing of the report, nor the decision to submit the paper for publication.

## Results

### Patient population and clinical outcomes

**Fig. 1:**
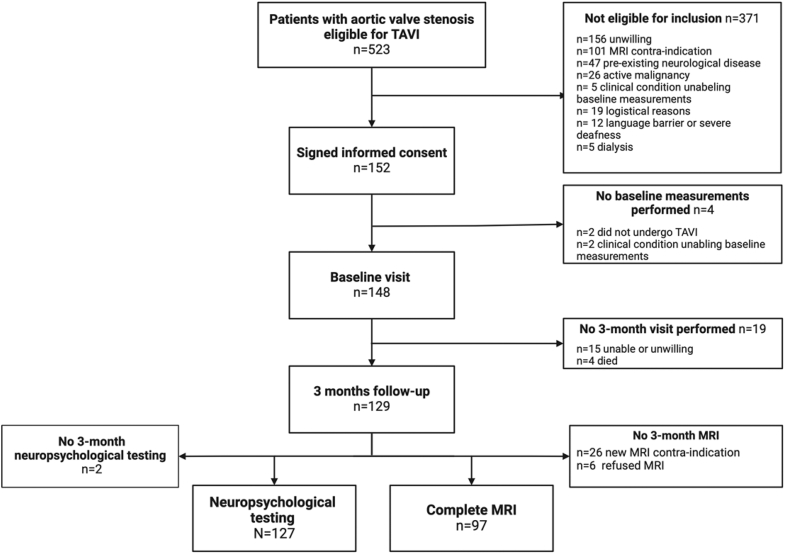
**Study flowchart**. Legend: TAVI: transcatheter aortic valve implantation; MRI: magnetic resonance imaging.

A total of 148 patients were included in this study and underwent TAVI between August 2020 to October 2022. Fig. 1 presents the study flowchart. Mean age was 80.5 ± 5.7 years, 43% were female, transfemoral access was used in 96.6%, and median STS-PROM (Society for Thoracic Surgeons Predicted Risk for 30-day Mortality) was 2.0% (IQR 1.4%–2.9%). Table 1 presents baseline patient characteristics. At baseline, mean aortic valve gradient was 45.6 ± 15.5 mmHg, peak aortic valve gradient 70.0 ± 22.3 mmHg, and left ventricular ejection fraction 51.9 ± 9.4%. At follow-up, 2.7% (n = 4) had died. Table 2 presents procedural characteristics and clinical outcomes. Supplementary Table S3 presents causes of death and other reasons for missed follow-up visits. Follow-up was complete in 1/3 patients with stroke (33%), 3/4 patients with TIA (75%), and 17/21 patients with delirium (81%). Median time between baseline and follow-up was 93 days (IQR 86–105) days. Cardiac output increased from 5.9 ± 1.4 to 6.3 ± 1.4 L/min (mean difference 0.37, 95% CI 0.12–0.62, p = 0.004) three months after TAVI. Cardiac index, i.e., cardiac output corrected for body surface area, increased from 3.1 ± 0.7 to 3.3 ± 0.8 L/min/m^2^, (mean difference 0.20, 95% CI 0.07–0.33, p = 0.004).

**Table 1:** Baseline characteristics of the study population.

**Demographics**	
Age (years)	80.5 ± 5.7
Female sex	63 (42.6%)
Body mass index (kg/m^2^)	27.8 ± 4.6
Body Surface Area (m^2^)	1.92 ± 0.19
New York Heart Association class ≥ III	68 (45.9%)
STS predicted risk of mortality (%)	2.0 (1.4–2.9)
EuroSCORE II (%)	2.4 (1.6–3.5)
Middle or high education^a^	91 (64.1%)
**Medical history**	
Atrial fibrillation	50 (33.8%)
Previous myocardial infarction	21 (14.2%)
Angina pectoris	27 (18.2%)
Significant coronary artery disease	46 (31.1%)
Previous percutaneous coronary intervention	26 (17.6%)
Previous coronary artery bypass graft	11 (7.4%)
Heart failure	21 (14.2%)
Hypertension	88 (59.5%)
Diabetes mellitus	32 (21.6%)
Previous cerebrovascular events	33 (22.3%)
Peripheral vascular disease	9 (6.1%)
Chronic obstructive pulmonary disease	20 (13.5%)
Obstructive sleep apnoea	10 (6.8%)
Malnutrition^b^	23 (15.5%)
Frailty^c^	12 (9.4%)
Cognitive impairment^d^	18 (12.4%)
Depression^e^	17 (11.9%)
Previous delirium	21 (14.2%)
**Physical examination at admission**	
Systolic blood pressure (mmHg)	140 ± 21
Diastolic blood pressure (mmHg)	73 ± 13
Mean arterial pressure (mmHg)	95 ± 13
Left bundle branch block	20 (13.5%)
Right bundle branch block	24 (16.2%)
Systolic annular area on CT (mm)	496 ± 89
Haemoglobin (mmol/L)	8.3 ± 5.3
Haematocrit (L/L)	0.39 ± 0.04
eGFR (mL/min/1.73 m^2^)	64.0 ± 16.5
**Echocardiography at admission**	
Mean gradient (mmHg)	45.6 ± 15.5
Peak gradient (mmHg)	70.0 ± 22.3
Aortic valve area (cm^2^)	0.89 ± 0.26
Left ventricular ejection fraction (%)	51.9 ± 9.4
Aortic regurgitation^f^	20 (13.6%)
Mitral regurgitation	39 (26.3%)
Tricuspid regurgitation	24 (16.5%)
Bicuspid aortic valve	16 (10.8%)
**Baseline MRI**	
Volume of white matter hyperintensities (mL)	13.1 (7.0–24.6)
Number of white matter hyperintensities	126 (32–216)
No white matter hyperintensities	5 (3.6%)
Fazekas 1	60 (42.9%)
Fazekas 2	55 (37.2%)
Fazekas 3	20 (13.5%)

STS: Society for Thoracic Surgeons; EuroSCORE: European System for Cardiac Operative Risk Evaluation; CT: computed tomography; eGFR: estimated Glomerular Filtration Rate.

a Verhage score ≥5

b Short Nutritional Assessment Questionnaire >1

c Edmonton frailty score >5

d Mini-mental state examination <24 or Montreal cognitive assessment <18

e Geriatric depression score >5

f Regurgitation moderate or severe.

**Table 2:** Procedural characteristics and clinical outcomes.

**Procedural characteristics**	
Transfemoral access	143 (96.6%)
Balloon-expandable valve	145 (98.0%)
Valve size	26 (23–26)
Annulus size (mm^2^)	496.0 ± 88.6
Local anaesthesia	142 (95.9%)
Pre dilatation	81 (54.7%)
Post dilatation	4 (2.7%)
Protamine given	141 (95.3%)
Procedural time (min)	50 (40–60)
Collagen-based closure device	143 (9.6%)
**Clinical outcomes at three months**	
All-cause mortality	4 (2.7%)
Cardiovascular mortality	3 (2.1%)
Periprocedural mortality (<30 days)	3 (2.1%)
Stroke	3 (2.1%)
Transient ischemic attack	4 (2.7%)
Delirium	21 (14.2%)
Re-hospitalization	11 (7.4%)
**Bleeding**	
Type 1 (minor)	23 (15.5%)
Type 2 (major)	1 (0.7%)
Type 3 (life-threatening)	9 (6.1%)
Type 4 (leading to death)	0
**Vascular complications**	
Pseudoaneurysm	5 (3.4%)
Closure device failure	16 (10.8%)
Requiring covered stent	2 (1.4%)
**Cardiac structural complications**	
Tamponade requiring pericardiocentesis	5 (3.4%)
Conversion to open surgery	1 (0.7%)
Permanent pacemaker implantation	27 (18.2%)
New onset left bundle branch block	27 (18.2%)
New-onset atrial fibrillation	14 (9.5%)
Acute kidney injury	2 (1.4%)
eGFR at discharge (mL/min/1.73 m^2^)	64.0 ± 17.0
Myocardial Infarction	2 (1.4%)
Valve thrombosis	3 (2.0%)
**Physical examination at three months**	
New York Heart Association class ≥ III	7 (4.7%)
Systolic blood pressure (mmHg)	155 ± 24
Diastolic blood pressure (mmHg)	78 ± 13
Mean arterial pressure (mmHg)	104 ± 14
**Paravalvular regurgitation at three months**	
No	61 (49.6%)
Trace/mild	53 (43.1%)
Moderate	8 (6.5%)
Severe	1 (0.8%)

eGFR: estimated Glomerular Filtration Rate.

### Brain MRI

**Fig. 2:**
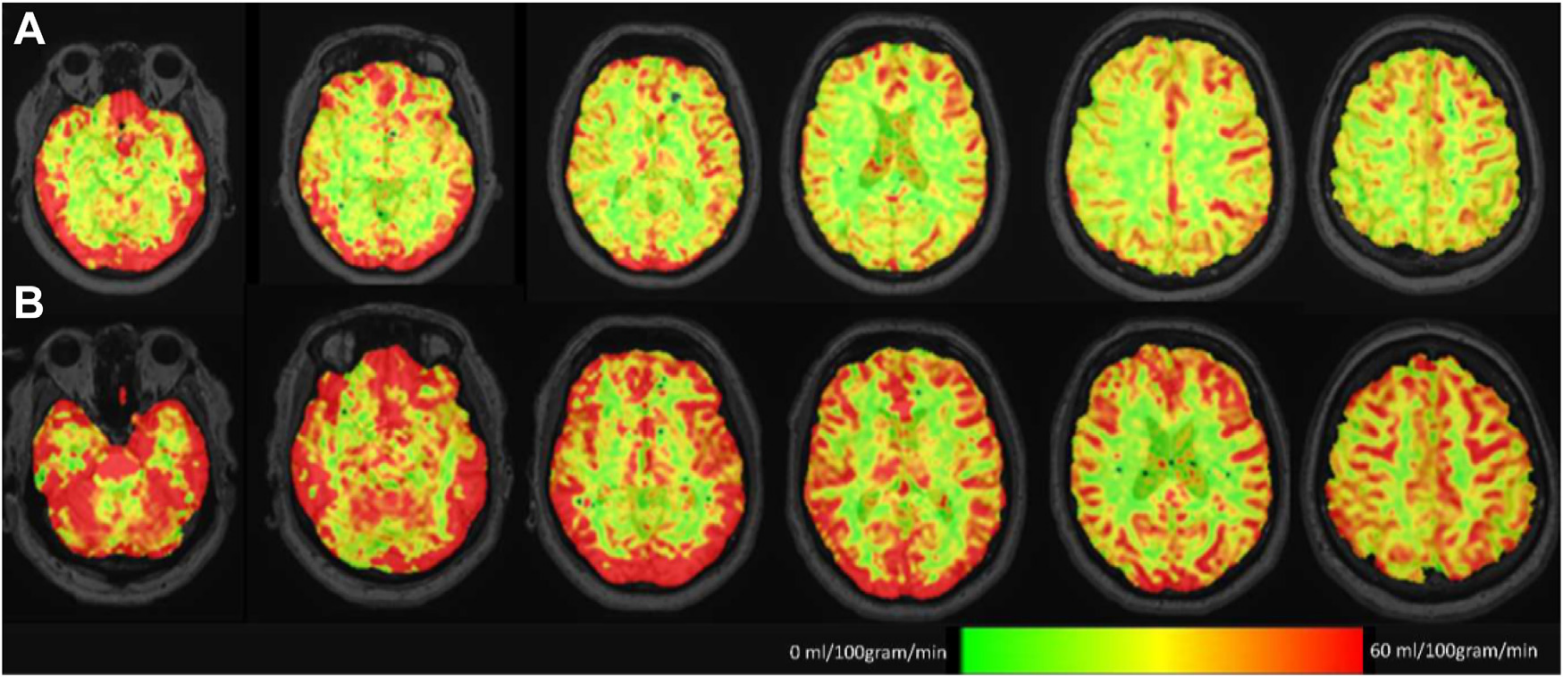
**Cerebral blood flow on arterial spin labelling magnetic resonance imaging**. Legend: Example of arterial spin labelling magnetic resonance imaging used to quantify cerebral blood flow. Areas of high blood flow are shown in red, while areas of lower blood flow are shown in green. The images illustrate an visual increase in cerebral blood flow between baseline (A) and three-month follow-up (B) scans.

**Fig. 3:**
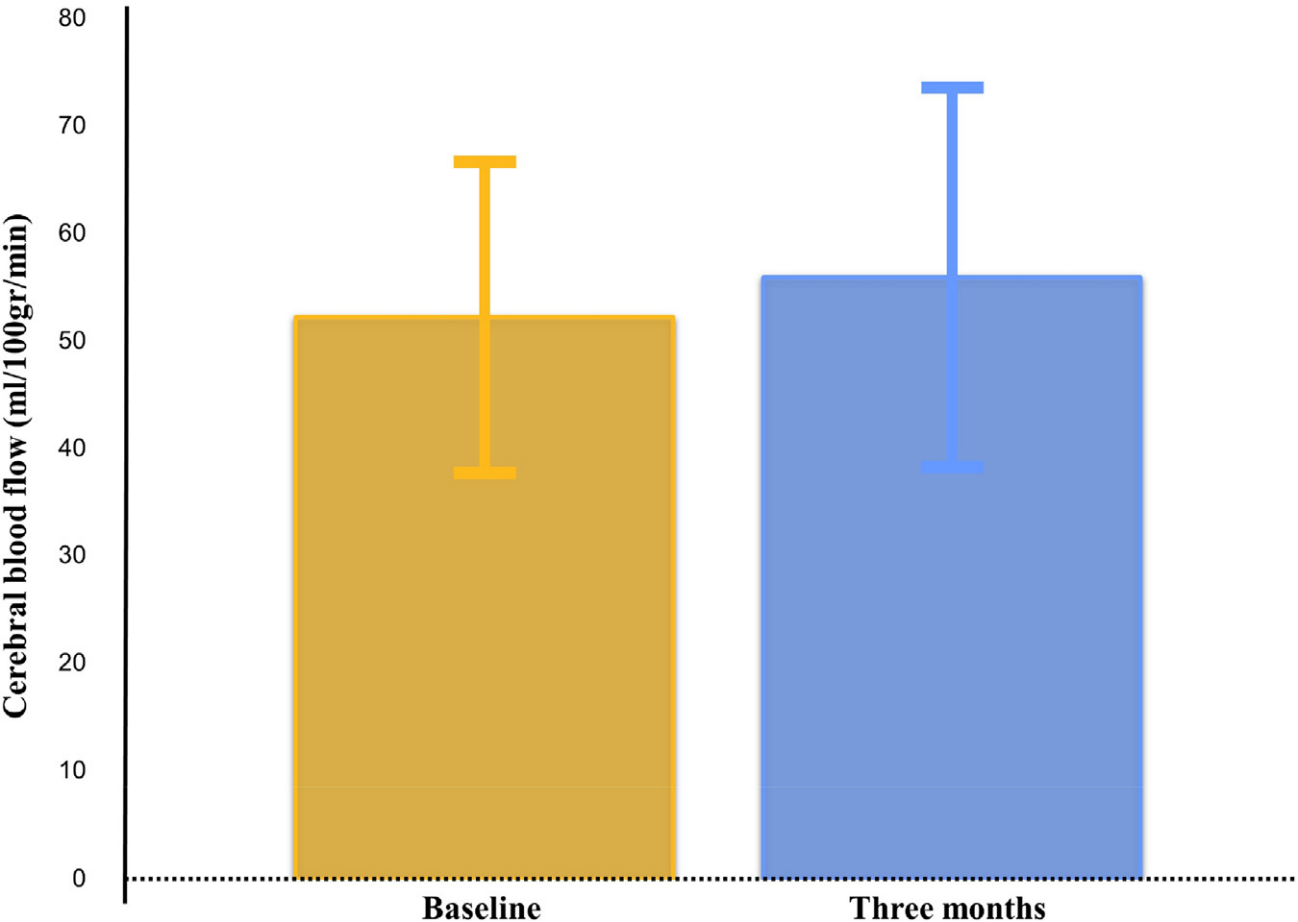
**Change in cerebral blood flow between baseline and three-month follow-up**. Legend: Increase in cerebral blood flow from baseline to three months after transcatheter aortic valve implantation.

A total of 97 (67%) patients underwent follow-up MRI. Follow-up MRI was not performed in 26 patients due to a new MRI contra-indication (e.g., permanent pacemaker implantation). Four patients died and 21 patients were otherwise unable or unwilling to undergo follow-up MRI. Supplementary Table S4 presents baseline characteristics in patients with and without follow-up MRI. Fig. 2 presents ASL-MRI image acquisition. Mean CBF at baseline was 52.2 ± 14.5 mL/100 g/min and mean CBF at three-month follow-up was 55.9 ± 17.7 mL/100 g/min (p = 0.005), Fig. 3. Mean increase in CBF was 3.8 mL/100 g/min (95% CI 1.15–6.36), corresponding to 8.9% increase. Increase in CBF was comparable between women and men (3.9 ± 13.2 vs 3.7 ± 12.2 mL/100 g/min, p = 0.94). Grey matter volumes, which are known to influence CBF, remained stable: 534.0 ± 59.7 vs 533.5 ± 62.7 mL (p = 0.68). Only 3.6% (n = 5) patients did not have baseline WMH; 42.9% (n = 60) had Fazekas 1 indicating punctate WMH lesions; 37.2% (n = 55) had Fazekas 2 with beginning confluence of WMH, and 13.5% (n = 20) had Fazekas 3 with large confluent WMH.^21^ Baseline WMH volume was 13.1 (IQR 7.0–24.6) mL and was distributed across 126 (IQR 32–216) lesions. At follow-up, WMH volume was 13.3 (IQR 5.8–21.5) mL in 164 (IQR 121–301) lesions (median increase was 0.1 mL, IQR 0.0–0.3 mL, p = 0.02). At three months, 9.5% patients (n = 9) had more than one mL increase in WMH.

### Cognitive functioning

**Fig. 4:**
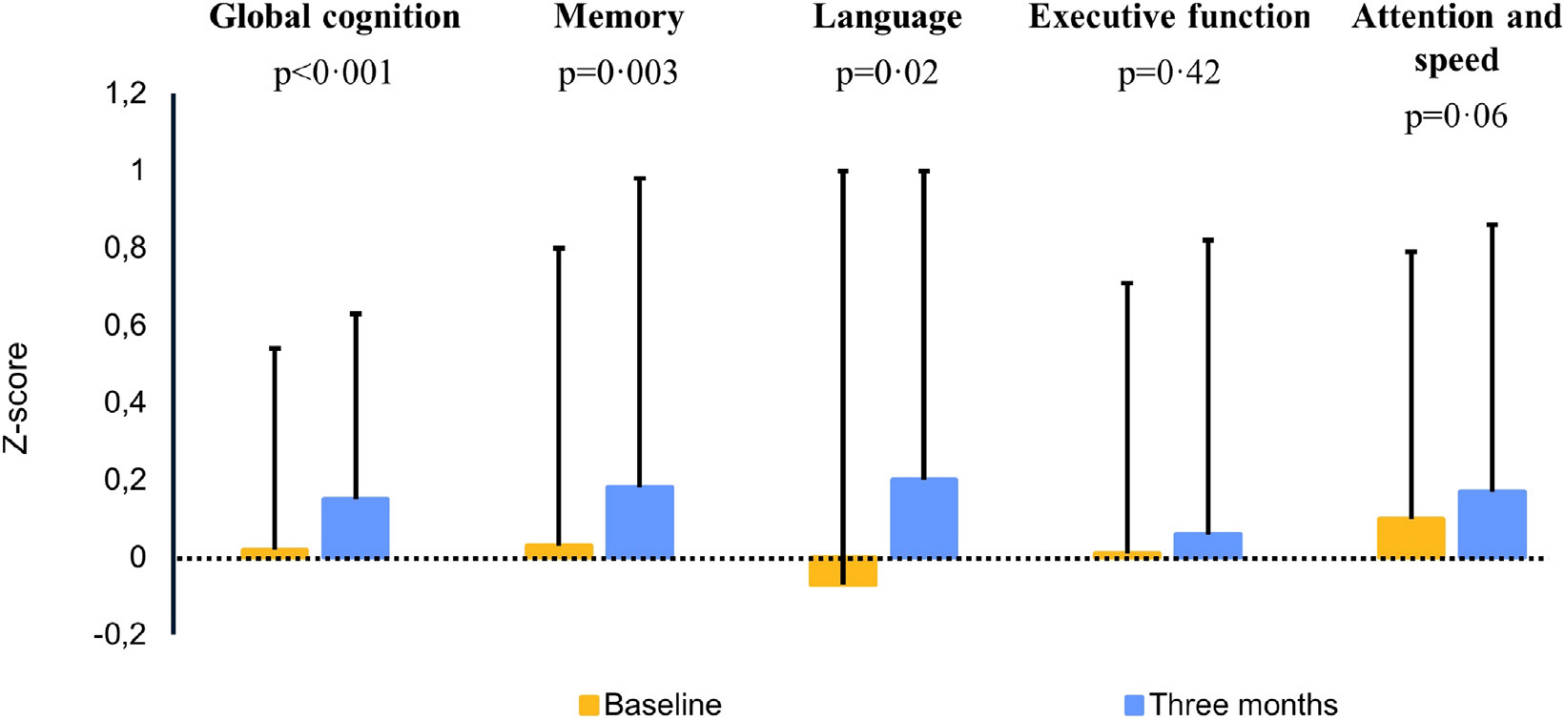
**Cognitive domain Z-scores for baseline and three-month follow-up**. Legend: Mean cognitive z-scores with standard deviation for global cognitive functioning and per domain at baseline and three months follow-up.

A total of 127 (88%) patients underwent follow-up neuropsychological assessment. MoCA scores improved from 23.3 ± 3.7 to 24.4 ± 3.3 (mean difference 1.1, 95% CI 0.6–1.6, p < 0.001) and MMSE scores from 26.4 ± 2.6 to 26.9 ± 2.4 (mean difference 0.5, 95% CI 0.1–9.9, p = 0.01). There was an overall increase in global cognitive z-scores (0.02 ± 0.52 to 0.15 ± 0.48, mean difference 0.13, 95% CI 0.06–0.20, p < 0.001). Table 3 presents the cognitive domain scores. Cognitive domains of memory (0.03 ± 0.77 to 0.18 ± 0.80, mean difference 0.16, 95% CI 0.05–0.26, p = 0.003) and language (−0.07 ± 1.07 to 0.20 ± 0.80, mean difference 0.27, 95% CI 0.05–0.48, p = 0.02) significantly improved, whereas executive function (0.01 ± 0.70 to 0.06 ± 0.76, mean difference 0.04, 95% CI −0.06 to 0.15, p = 0.42) and attention (0.10 ± 0.69 to 0.17 ± 0.69, mean difference 0.07, 95% CI −0.00 to 0.14, p = 0.06) did not change, Fig. 4. Quality of life improved from 66.4 ± 15.9 to 72.9 ± 12.8 (mean difference 6.5, 95% CI 3.9–9.1, p < 0.001). Raw cognitive test scores are displayed in Supplementary Table S5. Based on >0.5 SD change in overall cognitive functioning and lost to follow-up reasons, 22% (n = 30, mean Δ z-score: −0.39, 95% CI −0.49 to −0.29) of patients had a decline in cognitive functioning, 32% (n = 44, mean Δ z-score: 0.52, 95% CI 0.42–0.62) had improved cognition and 46% of the study population (n = 63, mean Δ z-score: −0.02, 95% CI −0.5 to 0.01) had stable cognition. Patients in the lowest tertile of baseline cognitive functioning, had the largest increase (n = 49), as compared to patients in the intermediate (n = 50) or highest tertile (n = 49) of (0.30, 95% CI 0.11–0.49, vs 0.55, 95% CI −0.00 to 0.18, vs 0.01–0.03–0.06, p = 0.002).

**Table 3:** Z-scores for cognitive functioning per domain at baseline and three-month follow-up.

	Baseline z-score	Follow-up z-score	Mean difference (95% confidence interval)	p-value
Global cognition	0.02 ± 0.52	0.15 ± 0.48	0.13 (−0.06–0.20)	<0.001
Attention	0.10 ± 0.69	0.17 ± 0.69	0.07 (−0.00-0.14)	0.06
Memory	0.03 ± 0.77	0.18 ± 0.80	0.16 (0.05–0.26)	0.003
Language	−0.07 ± 1.07	0.20 ± 0.80	0.27 (0.05–0.48)	0.02
Executive function	0.01 ± 0.70	0.06 ± 0.76	0.04 (−0.06–0.15)	0.42

Legend: Z-scores were constructed from raw test scores as: (test score–mean baseline score)/standard deviation. Z-scores from different neuropsychological tests are averaged into domain z-scores. Values are listed as mean ± standard deviation.

### Association between cardiac output, CBF, and cognitive functioning

A total of 95 (64%) patients had follow-up MRI and neuropsychological data available. Change in CBF was not associated with change in global cognitive functioning (B −0.0009, 95% CI −0.0073 to 0.0054), nor was there an association between change in cardiac output and change in CBF (B 0.7703, 95% CI −1.3973 to 2.9379) in a linear mixed model including age, sex, and education. Patients with cognitive decline, had a higher volume of new WMH than patients with stable or improved cognitive functioning (1.26 ± 2.96, vs 0.29 ± 0.67 mL, p = 0.03).

## Discussion

This prospective CAPITA study demonstrated increase in cerebral blood flow and overall global cognitive functioning in 148 patients at three months after TAVI. On a patient level, cognitive functioning at follow-up was stable in 46% of the patients, 32% of patients had improved cognitive functioning, and 22% a decline in cognitive functioning. Patients with cognitive decline had a larger volume of new WMH after TAVI. This is the first large scale study reporting on CBF in patients undergoing TAVI. Our results are consistent with two pilot studies (n = 31 and n = 15) suggesting that TAVI may increase CBF,^12^^,^^42^ but contradictory to a recent study (n = 42) that did not find improved CBF after TAVI.^43^

The brain receives about 12–15% of cardiac output.^13^^,^^44^ A decrease in cardiac output has a notably smaller effect on CBF than on peripheral blood flow: a 30% acute decrease in cardiac output correlates with only 10% reduction in CBF due to autoregulatory mechanisms.^13^ Severe aortic valve stenosis can negatively affect these mechanisms, which may be restored by TAVI. Vascular reactivity regulates vessel diameter in response to vasoactive stimuli, primarily paCO_2_.^44^ Hypocapnia and hyperoxia due to dyspnoea in patients with severe aortic valve stenosis can induce vasoconstriction. Relief of aortic valve stenosis by TAVI may normalize paCO_2_ and thereby improve CBF.^23^^,^^44^ Endothelial dysfunction plays a major role in the pathophysiology of aortic valve stenosis. Endothelial cells also regulate the vascular smooth muscle tone.^44^ Endothelial function can improve after TAVI.^45^, ^46^, ^47^ Improved endothelial function of the cerebrovascular vasculature following TAVI may increase CBF. Cerebral autoregulation ensures relatively constant CBF across a wide range of changes in blood pressure and body posture.^44^ Systemic blood pressure significantly increases immediately after valve implantation due to sudden afterload reduction in a usually hypertrophic left ventricle.^48^^,^^49^ To ensure follow-up in a new steady state, post procedural CBF was measured at three months.

We previously hypothesized that restoration of impaired cardiac output after TAVI would lead to improved CBF.^12^^,^^14^ Remarkably, there was no clear association between improvement in cardiac output and CBF. In addition to autoregulatory mechanisms, increased exercise capability, a perceived higher quality of life, and other lifestyle factors that improve after TAVI may contribute to higher CBF.^23^ Moreover, since this was an all-comers study including a heterogenous TAVI population, the relatively small sample size limited the option for subgroup analyses that may have cognitive benefit from increased cardiac output and CBF. CAPITA is the first larger scale study assessing new WMH beyond the early postprocedural period after TAVI. One in ten patients had more than 1 mL increase in WMH volume three months after TAVI. Previous small observational studies showed that three-quarters of patients undergoing TAVI had acute cerebral infarcts on early postprocedural MRI.^8^^,^^9^ However, these acute postprocedural cerebral lesions were diffusion weighted imaging lesions that usually resolve spontaneously within days to weeks and differ thereby substantially from WMH.^8^ In two previous studies, a quarter of these diffusion weighted imaging infarcts persisted as WMH.^9^^,^^10^ In addition, new WMH have also been reported at different locations than acute infarcts in longitudinal MRI studies.^9^^,^^10^ New WMH may negatively impact long term cognitive functioning due to their chronic nature. In addition, acute hemodynamic changes after TAVI may induce WMH by cerebral hypoperfusion during rapid pacing followed by hyperaemia directly after valve placement.^50^ These sudden hemodynamic changes can potentially damage vulnerable brains. In addition, many patients exhibit severe hypertension in the postprocedural days due to pre-existing left ventricular hypertrophy with sudden afterload reduction by TAVI.^48^^,^^49^ In fact, hypertension is one of the key risk factors resulting in WMH in non-TAVI patients and deserves more attention in the TAVI population.^26^ The CAPITA study demonstrated overall improvement in cerebral blood flow and cognitive functioning after TAVI, however with large difference between patients. It is reassuring that on an individual patient level, the majority of patients had preserved and or even improved cognitive functioning. Cognitive improvement was particularly found in patients with baseline cognitive impairment. Nevertheless, 22% of patients had cognitive decline. Previous studies on cognitive change after TAVI found similar results: approximately one third of patients had cognitive improvement after the procedure, and about ten percent of patients had cognitive decline.^3^, ^4^, ^5^^,^^11^^,^^51^ In contrast, only a few previous studies used a complete neuropsychological assessment covering multiple cognitive domains, whereas most used short screening tests, such as MoCA or MMSE, to evaluate cognitive functioning.^4^^,^^11^^,^^51^^,^^52^

In the current cohort, there was no association between change in CBF and cognitive functioning. Our study adds to the conflicting literature about the association between CBF and cognition in cardiovascular patients. A recent study of 42 patients undergoing TAVI demonstrated no change in cerebral blood flow, as measured with transcranial doppler, nor in cognitive functioning.^43^ In 27 patients undergoing cardiac surgery, CBF increased over a six-week period, but this increase did not correlate with change in cognitive functioning.^53^ Nor was there an association in patients with carotid artery disease.^54^ Studies are conflicting about a potential association in patients with heart failure.^22^^,^^44^^,^^54^^,^^55^ In the general population, there was no association between CBF and cognitive functioning,^54^^,^^56^ but lower cardiac output and CBF may predict for future, not current, dementia.^54^^,^^56^

Patients with cognitive decline had a larger volume of new WMH on follow-up brain MRI. In general, there is ample evidence on the association between WMH and cognitive dysfunction.^57^ In patients with severe aortic valve stenosis scheduled for TAVI, increased WMH volumes were associated with impaired baseline cognitive functioning.^58^ Moreover, baseline WMH have been identified as a predictor for postprocedural silent brain infarcts.^9^ Only one previous study of 28 patients assessed new WMH after TAVI and also found an association with decrease in MMSE scores.^10^ We hypothesize that WMH may be an important and underrepresented biomarker for cognitive decline after TAVI, and future studies should identify risk factors for new WMH.

CAPITA systematically addressed cardiac output, CBF, WMH, and cognitive functioning. It adds to the literature that TAVI has the potential to improve cardiac output, CBF and cognitive functioning in a subset of patients. Cognitive improvement following TAVI is probably multifactorial and a result of overall improved quality of life; a more active lifestyle including social interactions; improved exercise capability due to relieve of aortic valve stenosis; the absence of new postprocedural brain infarcts, and partly of improved CBF. The current study reassures that TAVI is safe for cognitive functioning in most patients. Potentially, TAVI may slow cognitive decline in patients with pre-existing cognitive impairment.

On the other hand, one in five patients had cognitive decline. These patients had a higher volume of new WMH at three months. WMH are potentially more detrimental to cognitive functioning than was previously assumed and should be further investigated. Instead of studies that focus on short term acute infarcts, future MRI studies should implement a longer term follow-up including WMH.

Strengths of the study include the large number of patients undergoing TAVI with baseline and follow-up measurements. The use of a standardized neuropsychological test battery allowed to look at specific cognitive domains. However, the observational design has its inherent limitations. Patients with permanent pacemaker implantation could not undergo follow-up MRI. Although baseline characteristics of patients who could not undergo follow-up MRI were in essence comparable to patients without follow-up MRI, we cannot exclude that the reason to miss a follow-up assessment was associated with decreased CBF or cognitive functioning, although we included patients who died or missed a follow-up visit due to a presumable cause that affects CBF or cognitive function in the cognitive decline group. ASL-MRI was performed with fixed transit times, which is the estimated time blood needs to travel from the carotid artery to the cerebral circulation.^22^ Elderly patients with arterial stiffness may require different transit times, however, as the primary outcome measure was change in CBF, fixed transit times will not affect this outcome.^22^ The current study only included patients who underwent TAVI and no controls, as we believe it is not ethical to delay TAVI in patients with a treatment indication. CBF demonstrates a gradual age-related 0.5% decrease per year and spontaneous improvement in CBF would not be expected in this 80-year-old population.^22^^,^^44^ Similarly, spontaneous cognitive improvement is usually unexpected in the elderly. However, we cannot exclude regression to the mean in absence of a control group, nor were representative normative mean test scores available. A parallel version of the verbal learning test was used to reduce any potential learning effects, but we cannot completely rule out learning effects for the other tests. Similarly, potential test-retest bias cannot be ruled out. A proportion of the approached eligible patients did not want to participate in the study due to presumed burden of the study visits, therefore our results cannot be directly extrapolated to the frailest patients. Since this was a single-centre study, our results may not be directly extrapolatory to other TAVI centres, in particular those performed under general anaesthesia. Lastly, the study was performed during the COVID-19 pandemic, which has potentially influenced patients’ decisions to take part in the study or attend follow-up visits.

In conclusion, this prospective study of patients with severe aortic valve stenosis undergoing TAVI demonstrated that overall cardiac output, CBF, and cognitive functioning improved at three months after TAVI. Cognitive functioning was stable in 46%, improved in 32%, and declined in 22% of patients following TAVI. Patients with worst baseline cognitive functioning showed the greatest cognitive improvement. There was no association between change in cardiac output, CBF, and cognitive functioning. Patients with cognitive decline had a larger volume of new WMH on follow-up MRI.

## Contributors

AN: conceptualization, data curation, data access and verification, formal analysis, funding acquisition, investigation, methodology, project administration, visualization, writing (original draft); KH: data curation, data access and verification formal analysis, investigation, project administration, validation, visualization, writing (original draft, review and editing); AL: conceptualization, data curation, formal analysis, investigation, methodology, resources, validation, writing (review and editing); CM: data curation, formal analysis, investigation, resources, writing (review and editing); MD: conceptualization, methodology, validation, writing (review and editing); EE: data curation, formal analysis, investigation, software, resources, writing (review and editing); JM: data curation, formal analysis, methodology, validation, writing (review and editing); AS: data curation, formal analysis, methodology, resources, software, writing (review and editing); BB: data curation, formal analysis, investigation, methodology, resources, software, supervision, validation, writing (review and editing); WF: conceptualization, investigation, methodology, resources, validation, writing (review and editing); JB: investigation, resources, supervision, writing (review and editing); JP: supervision, writing (review and editing); GB: conceptualization, funding acquisition, investigation, methodology, supervision, validation, writing (editing); RD: conceptualization, data curation, data access and verification, funding acquisition, investigation, methodology, project administration, resources, supervision, validation, writing (original draft, review and editing).

## Data sharing statement

Data collected for this study are available from the corresponding author upon reasonable request. Data can only be shared when requests comply with the General Data Protection Regulation.

## Declaration of interests

Dr. Delewi received educational fees from Boston Scientific and Edwards Lifesciences. Prof. dr. Majoie received funds from CVON/Dutch Heart Foundation, Stryker, European Commission, and Healthcare Evaluation Netherlands (unrelated to this project; all paid to institution) and is (minority interest) shareholder of Nicolab. Dr. Moonen is appointed at NCDC, which is funded in the context of Deltaplan Dementie from ZonMW Memorabel (projectnr 73305095005) and Alzheimer Nederland. Prof. J.J. Piek is a consultant for Philips. Prof. Dr. Biessels, prof. dr. van de Flier, and dr. Bron received a Dutch Heart Foundation (CVON 2018-28 & 2012-06 Heart Brain Connection) grant. Dr. Bron received research funding from Medical Delta, Health ∼ Holland, Erasmus MC, ZonMW and the European Commission. Prof. Dr. van der Flier received research funding from by ZonMW, NWO, EU-FP7, EU-JPND Alzheimer Nederland, Hersenstichting CardioVascular Onderzoek Nederland, Health ∼ Holland, Topsector Life Sciences & Health, stichting Dioraphte, Gieskes-Strijbis fonds, stichting Equilibrio, Edwin Bouw fonds, Pasman stichting, Alzheimer & Neuropsychiatrie Foundation, Philips, Biogen MA Inc, Novartis-NL, Life-MI, AVID, Roche BV, Fujifilm, Eisai, Combinostics. The other authors report no disclosures.
