# Supplementary material for: Cerebral blood flow and cognitive functioning in patients undergoing transcatheter aortic valve implantation
Source: eClinicalMedicine. 2025 Feb 14;81:103092. doi: 10.1016/j.eclinm.2025.103092 (PMC11872408; doi:10.1016/j.eclinm.2025.103092)
Supplement: Supplementary Tables [file mmc1.docx]

**Supplemental material - content**

Supplementary methods

Supplementary Table S1: Inclusion and exclusion criteria for the study

Supplementary Table S2. Overview of cognitive tests used in the neuropsychological assessment

Supplementary Table S3: Baseline patient characteristics of patients with and without follow-up MRI

Supplementary Table S4: Neuropsychological test scores

Supplementary Table S5: Causes of death and loss to follow-up

Supplementary references

**Supplementary methods**

**Sample size**

Based on our pilot data,^1^ we hypothesize a 10±12% increase in CBF after TAVI. With 80% power and alpha of 0.05, the required sample size is 126. If a TAVI procedure is complicated by the need for permanent pacemaker implantation, subsequent MRI follow-up will be contra-indicated. In addition, some patients will not be able to take part in all follow-up measurements due to death, concomitant disease, or institutionalization. Therefore, with an expected 21% of patients who are not able to undergo follow-up MRI, a total of 152 patients will be needed.

**Supplementary Table S1. Inclusion and exclusion criteria for the study**

| Inclusion criteria | |
| --- | --- |
| 1 | Presence of severe symptomatic aortic valve stenosis and eligible for TAVI |
| 2 | Able and willing of giving informed consent |
| 3 | Age > 18 years |
| Exclusion criteria | |
| 1 | Presence of a MRI contra-indication |
| 2 | Known neurological disease, excluding cerebrovascular events without sequelae |
| 3 | Active malignant disease |
| 4 | Insufficient mastery of the Dutch language |
| 5 | Non-atherosclerotic vascular disease (e.g., vasculitis) |
| 6 | Dialysis treatment for renal failure |
| 7 | Planned surgery with general anaesthesia within three months after TAVI |

MRI: magnetic resonance imaging, TAVI: transcatheter aortic valve implantation.

**Supplementary Table S2. Overview of cognitive tests used in the neuropsychological assessment**

| Cognitive domain | Cognitive tests |
| --- | --- |
| Memory | 15-word-auditory verbal learning test: total immediate recall, delayed recall, and recognition score^a, 2^ Visual association test: part A^3^ |
| Language | Visual association test: naming^3^ |
| Attention and psychomotor speed | Trail making test: part A^b, 4^  Stroop color word test: card I and II^5 #^ Letter Digit Substitution Test^6^ Digit Span: forward condition^7^ |
| Executive functioning | Trail making test: index score of part B/A^4^ Stroop color word test: interference score^c, 5^ Digit Span: backward condition ^7^ |
| Global cognition | Mean score of 4 domains |
| Global cognitive screening | Montreal Cognitive Assessment^8^  Mini-Mental State Examination^9^ |
| Depressive symptoms | Geriatric Depression Scale^10^ |
| Apathy symptoms | Starkstein Apathy Scale^11^ |
| EuroQol-5D incl Visual Analog Scale | Health-related quality of life^12^ |

a. In order to minimize test-retest effects, the 15 word test was used with different words at 3 months follow-up than at baseline.

b. Z-scores were inverted (z*-1), because higher test scores indicate worse cognitive functioning

c. Stroop color word test interference score is calculated as card III/([card I + card II] / 2)

**Supplementary Table S3. Causes of death and loss to follow-up**

| Causes of death | |
| --- | --- |
| 1* | Asphyxiation |
| 2* | Sudden cardiac death |
| 3* | Suicide |
| 4* | End stage heart failure |
| Causes of missed follow-up | |
| 5* | Permanent pacemaker implantation, refused follow-up neuropsychological assessment due to admission in rehabilitation unit |
| 6* | Permanent pacemaker implantation, refused follow-up neuropsychological assessment after transient ischemic attack |
| 7 | Permanent pacemaker implantation, refused follow-up neuropsychological assessment |
| 8 | Permanent pacemaker implantation, refused follow-up neuropsychological assessment |
| 9* | Admission to nursing home, too unwell to travel to Amsterdam UMC |
| 10* | Nursing home admission due to major cerebral infarct |
| 11* | Multiple cerebral infarcts after the procedure, persistent delirium and therefore unable to visit Amsterdam UMC |
| 12* | Moved abroad without notice and therefore lost to follow-up. Upon return (1 year later) diagnosed with dementia by general practitioner and unable to travel to Amsterdam UMC |
| 13* | Too unwell, admission to nursing home, died after 6 months after TAVI |
| 14* | Too unwell, admission to nursing home, died after 4 months after TAVI |
| 15 | Multiple times no show, eventually refused follow-up |
| 16 | Refused all follow-up measurements |
| 17 | Refused all follow-up measurements |
| 18 | Refused all follow-up measurements |
| 19 | Refused all follow-up measurements |

***** considered as potentially associated with cognitive decline and therefore reported as such.

**Supplementary Table S4: Baseline patient characteristics of patients with and without follow-up MRI.**

| Demographics | With 3-month MRI (n=97) | Without 3-month MRI (n=51) | p-value |
| --- | --- | --- | --- |
| Age (years) | 81.0±5.7 | 79.6±5.6 | 0.13 |
| Female sex | 43 (44%) | 20 (39%) | 0.55 |
| Body mass index (kg/m^2^) | 27.6±4.8 | 28.1±4.2 | 0.48 |
| Body Surface Area (m^2^) | 1.90±0.19 | 1.95±0.18 | 0.13 |
| New York Heart Association class III/IV | 38 (39%) | 30 (59%) | 0.02 |
| STS predicted risk of mortality (%) | 2.1 (1.4-3.0) | 1.9 (1.3-2.5) | 0.22 |
| EuroSCORE II (%) | 2.5 (1.6-3.7) | 2.4 (1.6-3.3) | 0.64 |
| Middle or high education^a^ | 56 (60%) | 35 (71%) | 0.19 |
| Medical history |  |  |  |
| Atrial fibrillation | 34 (35%) | 16 (31%) | 0.65 |
| Previous myocardial infarction | 13 (13%) | 8 (16%) | 0.71 |
| Angina pectoris | 17 (18%) | 10 (20%) | 0.76 |
| Significant coronary artery disease | 29 (30%) | 17 (33%) | 0.67 |
| Previous percutaneous coronary intervention | 18 (19%) | 8 (16%) | 0.66 |
| Previous coronary artery bypass graft | 6 (6%) | 5 (10%) | 0.43 |
| Heart failure | 12 (12%) | 9 (18%) | 0.38 |
| Hypertension | 58 (60%) | 30 (59%) | 0.91 |
| Diabetes mellitus | 17 (18%) | 15 (29%) | 0.10 |
| Previous cerebrovascular events | 20 (21%) | 13 (26%) | 0.50 |
| Peripheral vascular disease | 6 (6%) | 3 (6%) | 0.94 |
| Chronic obstructive pulmonary disease | 7 (7%) | 7 (14%) | 0.20 |
| Obstructive sleep apnoea | 6 (6%) | 4 (8%) | 0.70 |
| Malnutrition^b^ | 7 (8%) | 9 (18%) | 0.06 |
| Frailty^c^ | 9 (11%) | 3 (7%) | 0.41 |
| Cognitive impairment^d^ | 13 (14%) | 5 (10%) | 0.52 |
| Depression^e^ | 11 (12%) | 6 (12%) | 0.97 |
| Previous delirium | 6 (6%) | 3 (6%) | 0.99 |
| Physical examination |  |  |  |
| Systolic blood pressure (mmHg) | 141±22 | 139±19 | 0.53 |
| Diastolic blood pressure (mmHg) | 73±13 | 72±13 | 0.73 |
| Mean arterial pressure (mmHg) | 96±13 | 95±13 | 0.57 |
| Left bundle branch block | 15 (16%) | 5 (10%) | 0.34 |
| Right bundle branch block | 12 (12%) | 12 (24%) | 0.08 |
| Systolic annular area on CT (mm) | 488±84 | 510±95 | 0.45 |
| Haemoglobin (mmol/L) | 8.0±0.9 | 7.7±0.9 | 0.10 |
| Haematocrit (L/L) | 0.39±0.04 | 0.38±0.04 | 0.09 |
| eGFR (mL/min/1.73m2) | 65.3±15.7 | 61.6±18.0 | 0.19 |
| Echocardiography at admission |  |  |  |
| Mean gradient (mmHg) | 44.3±15.7 | 48.0±15.2 | 0.18 |
| Peak gradient (mmHg) | 68.0±21.9 | 73.7±22.9 | 0.14 |
| Aortic valve area (cm^2^) | 0.91±0.29 | 0.86±0.20 | 0.33 |
| Left ventricular ejection fraction (%) | 52.1±8.8 | 51.7±10.2 | 0.84 |
| Aortic regurgitation ^f^ | 11 (11%) | 9 (18%) | 0.29 |
| Mitral regurgitation | 27 (28%) | 12 (24%) | 0.57 |
| Tricuspid regurgitation | 18 (19%) | 6 (12%) | 0.30 |
| Bicuspid aortic valve | 12 (13%) | 4 (8%) | 0.44 |

a. Verhage score ≥5; b. Short Nutritional Assessment Questionnaire >1; c. Edmonton frailty score >5; d. Mini-mental state examination <24 or Montreal cognitive assessment <18; e. Geriatric depression score >5; f. Regurgitation moderate or severe. CT: computed tomography; eGFR: estimated Glomerular Filtration Rate; EuroSCORE: European System for Cardiac Operative Risk Evaluation; MRI: magnetic resonance imaging; STS: Society for Thoracic Surgeons.

**Supplementary Table S5. Raw test scores and standardized z-scores for baseline and follow-up neuropsychological testing**

|  | Baseline raw test score | Follow-up raw test score | Delta raw test score | Baseline z-score | Follow-up z-score | Delta z-score | p-value |
| --- | --- | --- | --- | --- | --- | --- | --- |
| Global cognition  (mean of domains scores) | - | - | - | 0.02±0.52 | 0.15±0.49 | 0.13±0.37 | <0.001 |
| Attention  Digit span forward  Letter-Digit Substitution Test  Trail Making Test A^a^  Mean of Stroop I and II^a^ | 7.6±1.8  35.6±9.1  56.4±23.4  61.2±12.6 | 7.6±1.7  36.2±9.2  54.2±20.8  60.8±14.2 | 0.1±1.5  0.6±4.4  -2.3±18.4  -0.4±8.7 | 0.10±0.69  0.08±0.99  0.10±0.96  0.15±0.93  0.13±0.88 | 0.17±0.69  0.13±0.97  0.16±0.97  0.24±0.82  0.16±0.99 | 0.07±0.37  0.05±0.83  0.07±0.46  0.09±0.73  0.03±0.61 | 0.06  0.56  0.15  0.20  0.63 |
| Memory  RAVLT immediate recall  RAVLT delayed recall  RAVLT recognition  Visual association test A *Visual association test B*^b^ | 32.3±9.5  5.8±3.3  27.1±2.9  11.5±1.3  10.3±2.2 | 35.1±9.8  6.6±3.3  27.4±2.8  11.5±1.5  10.3±2.4 | 2.8±7.7  0.8±2.5  0.2±2.5  -0.0±1.3  -0.0±2.0 | 0.03±0.77  0.03±1.00  0.04±1.04  0.00±1.00  0.04±1.01 | 0.18±0.80  0.32±1.03  0.28±1.06  0.08±0.95  0.06±0.88 | 0.15±0.54  0.29±0.81  0.24±0.79  0.08±0.85  -0.03±0.90 | 0.003  <0.001  0.002  0.35  0.77  0.96 |
| Language  Visual association test naming | 11.8±0.4 | 11.9±0.3 | 0.1±0.4 | -0.07±1.07  -0.07±1.07 | 0.20±0.80  0.20±0.80 | 0.27±1.12  0.27±1.12 | 0.02  0.02 |
| Executive function  Digit span backward  Trail making Test B/A index ^b^  *Trail making Test B*^a,b^  Stroop interference ^b^  *Stroop I*^a,b^  *Stroop II*^a,b^  *Stroop III,*^ab^ | 4.8±1.5  2.8±0.9  151.4±63.6  2.19±0.44  53.1±10.8  69.2±16.7  133.3±36.3 | 4.9±1.5 2.8±1.0  144.1±63.1  2.17±0.55  54.3±16.3  67.2±15.3  131.1±42.6 | 0.05±1.36  -0.0±1.0  -7.3±45.1  -0.02±0.52  1.2±12.1  -2.0±10.5  -2.3±32.3 | 0.01±0.70  0.06±1.00  0.03±0.88  -  0.03±0.89  -  -  - | 0.06±0.76  0.10±1.05  0.07±0.95  -  0.07±1.12  -  -  - | 0.04±0.58  0.03±0.93  0.04±0.92  -  0.04±1.05  -  -  - | 0.42  0.72  0.65  0.10  0.68  0.29  0.05  0.47 |
| Questionnaires  Quality of life - EQ5D  Visual analogue scale  Geriatric depression scale  Starkstein apathy scale | 2 (1-3)  66.4±15.9  2.3±2.3  11.3±5.3 | 2 (0-2)  72.9±12.8  2.0±2.4  10.9±4.9 | 0  6.5±13.7  -0.3±2.1  -0.4±4.0 | -  -  -  - | -  -  -  - | -  -  -  - | 0.11  <0.001  0.17  0.27 |
| Screening  MoCA  MMSE | 23.3±3.7  26.4±2.6 | 24.4±3.3  26.9±2.4 | 1.1±2.8  0.6±2.1 | -  - | -  - | -  - | <0.001  0.01 |

a. Not included in calculation of domain scores. b. Z-scores were inverted (z*-1), because higher test scores indicate worse cognitive functioning
Stroop color word test interference is calculated as card III/([card I + card II] / 2).
MoCA: Montreal Cognitive Assessment; MMSE: mini-mental state examination; RAVLT: Rey Auditory Verbal learning Test

**References**

1. Vlastra W, van Nieuwkerk AC, Bronzwaer ASGT, *et al.* Cerebral Blood Flow in Patients with Severe Aortic Valve Stenosis Undergoing Transcatheter Aortic Valve Implantation. *J Am Geriatr Soc* 2021; **69**: 494–9.
2. Saan R, Deelman B. De 15‐woordentest A en B (een voorlopige handleiding). Groningen, The Netherlands: AZG: Afdeling Neuropsychologie; 1986.
3. Lindeboom J, Schmand B, Tulner L, Walstra G, Jonker C. Visual association test to detect early dementia of the Alzheimer type. J Neurol Neurosurg Psychiatry. 2002 Aug;73(2):126-33.
4. Reitan RM. Validity of the Trail Making Test as an indicator of organic brain damage. Perceptual and motor skills. 1958 8.3:271-276.
5. Van der Elst W, Van Boxtel MP, Van Breukelen GJ, Jolles J. The Stroop color-word test: influence of age, sex, and education; and normative data for a large sample across the adult age range. Assessment. 2006 Mar;13(1):62-79.
6. van der Elst W, van Boxtel MP, van Breukelen GJ, Jolles J. The Letter Digit Substitution Test: normative data for 1,858 healthy participants aged 24-81 from the Maastricht Aging Study (MAAS): influence of age, education, and sex. J Clin Exp Neuropsychol. 2006 Aug;28(6):998-1009.
7. Lindeboom J, Matto D. Cijferreeksen en Knox blokken als concentratietests voor ouderen [Digit series and Knox cubes as concentration tests for elderly subjects]. Tijdschr Gerontol Geriatr. 1994 May;25(2):63-8.
8. Nasreddine ZS, Phillips NA, Bédirian V, Charbonneau S, Whitehead V, Collin I, Cummings JL, Chertkow H. The Montreal Cognitive Assessment, MoCA: a brief screening tool for mild cognitive impairment. J Am Geriatr Soc. 2005 Apr;53(4):695-9.
9. Folstein MF, Folstein SE, McHugh PR. "Mini-mental state". A practical method for grading the cognitive state of patients for the clinician. J Psychiatr Res. 1975 Nov;12(3):189-98.
10. Almeida OP, Almeida SA. Short versions of the geriatric depression scale: a study of their validity for the diagnosis of a major depressive episode according to ICD-10 and DSM-IV. Int J Geriatr Psychiatry. 1999 Oct;14(10):858-65.
11. Starkstein SE, Mayberg HS, Preziosi TJ, Andrezejewski P, Leiguarda R, Robinson RG. Reliability, validity, and clinical correlates of apathy in Parkinson's disease. J Neuropsychiatry Clin Neurosci. 1992 Spring;4(2):134-9.
12. The EuroQol Group. EuroQol*—a newfacility for the measurement of health-related quality of life. Health Policy (New York) 1990; 16:199–206.
